# *Arabidopsis* CNGC Family Members Contribute to Heavy Metal Ion Uptake in Plants

**DOI:** 10.3390/ijms20020413

**Published:** 2019-01-18

**Authors:** Ju Yeon Moon, Célestine Belloeil, Madeline Louise Ianna, Ryoung Shin

**Affiliations:** 1 RIKEN Center for Sustainable Resource Science, 1-7-22 Suehirocho, Tsurumi-ku, Yokohama, Kanagawa 230-0045, Japan; juyeon.moon@riken.jp (J.Y.M.); celestine.belloeil@gmail.com (C.B.); mianna@myune.edu.au (M.L.I.); 2 Université Paris Diderot, 5 rue Thomas Mann, 75013 Paris, France; 3 School of Science and Technology, UNE, Armidale, New South Wales 2351, Australia

**Keywords:** *Arabidopsis*, cadmium, cyclic nucleotide-gated channel (CNGC), ion uptake, lead

## Abstract

Heavy metal ions, including toxic concentrations of essential ions, negatively affect diverse metabolic and cellular processes. Heavy metal ions are known to enter cells in a non-selective manner; however, few studies have examined the regulation of heavy metal ion transport. Plant cyclic nucleotide-gated channels (CNGCs), a type of Ca^2+^-permeable-channel, have been suggested to be involved in the uptake of both essential and toxic cations. To determine the candidates responsible for heavy metal ion transport, a series of *Arabidopsis* CNGC mutants were examined for their response to Pb^2+^ and Cd^2+^ ions. The primary focus was on root growth and the analysis of the concentration of heavy metals in plants. Results, based on the analysis of primary root length, indicated that AtCNGC1, AtCNGC10, AtCNGC13 and AtCNGC19 play roles in Pb^2+^ toxicity, while AtCNGC11, AtCNGC13, AtCNGC16 and AtCNGC20 function in Cd^2+^ toxicity in *Arabidopsis*. Ion content analysis verified that the mutations of AtCNGC1 and AtCNGC13 resulted in reduced Pb^2+^ accumulation, while the mutations of AtCNGC11, AtCNGC15 and AtCNGC19 resulted in less Pb^2+^ and Cd^2+^ accumulation in plants. These findings provide functional evidence which support the roles of these AtCNGCs in the uptake and transport of Pb^2+^ or Cd^2+^ ion in plants.

## 1. Introduction

Toxic heavy metals (e.g., cadmium (Cd), lead (Pb), strontium (Sr), or mercury (Hg)), as byproducts of rapidly growing industries, have been released into and accumulated in the soils at many sites, and have exerted adverse impacts on ecosystems [1,2,3]. Their detrimental effects have been attributed to their competition with other essential cations for binding to enzymes, inhibition of enzyme activity, or inducing the overproduction of reactive oxygen species (ROS), which leads to oxidative stress and potential cell death [3,4,5]. A number of other heavy metals (including iron (Fe), copper (Cu), zinc (Zn), and nickel (Ni)), however, function as essential micronutrients in a variety of metabolic and cellular processes, such as primary/secondary metabolism, gene regulation, signal transduction, and hormone perception [6]. Essential metal ions, such as Zn^2+^ and Fe^2+^, are redox-active based and are involved in oxidation/reduction processes. An overabundance of these metals in cells, however, induces ROS formation. An excess of essential metals, as well as non-essential metals (e.g., Cd or Pb), has an adverse effect on animal and plant cells. Therefore, organisms must maintain a fine-tuned homeostasis of the levels of heavy metals within a cell or have a mechanism that regulates their transport into/out of cells. However, the mechanism by which metal ions enter into plant and animal cells is relatively obscure. Non-essential toxic heavy metals can be non-selectively taken up by organisms largely because of their irrelevance to any known cellular function. Interestingly, some heavy metals, such as cesium (Cs) and Sr have physicochemical properties that are similar to the essential minerals potassium (K) and calcium (Ca) [7,8,9,10,11]. It has been shown that the radionuclide Cs likely enters plant cells through the K transport system [11]. 

Cyclic nucleotide-gated ion channels (CNGCs) have been suggested as potential candidates of ligand-gated/voltage-independent/cation transporters that play a role in the plant response to biotic/abiotic stresses, growth and development, and ion homeostasis [12,13,14,15]. CNGCs are activated (gated) by direct binding of cyclic nucleotides (CNs), such as cAMP (cyclic adenosine monophosphate) and cGMP (cyclic guanosine monophosphate), as well as by diverse molecules and ions, like Ca^2+^, K^+^, and Na^+^, and can be inactivated by binding Ca^2+^-activated calmodulin (CaM) in a feedback mechanism [16,17,18]. In plants, CNGCs and Shaker-type K^+^-selective ion channels (voltage-dependent) have been postulated to be major targets of these ligands due to sharing some sequence homology and similarity in secondary structure. CN binding domains (CNBDs) are mainly present in the C-termini of plant CNGCs and Shaker-type K^+^ channels coinciding with a CaM binding domain [19,20]. Despite their high similarity to Shaker-type voltage-gated channels, the heterologous expression of plant CNGCs in various yeast mutants deficient in ion uptake or efflux have demonstrated that CNGCs have a lower ion selectivity than Shaker-type channels and lower permeability to monovalent and divalent cations (such as K^+^, Na^+^, and Ca^2+^) across the plasma membrane [9,21,22,23,24]. After the first identification of a plant CNGC, barley HvCBT1 (*Hordeum vulgare* CaM-binding transporter), that binds to CaM in the C-terminus in a Ca^2+^-dependent manner [23], AtCNGC1 and AtCNGC2 were identified in *Arabidopsis*, and NtCBP4 was identified in tobacco (*Nicotiana benthamiana*). All were predicted to have a CaM-binding site at the CNBD in these CNGCs [25,26,27,28]. The *Arabidopsis* genome contains 20 CNGC family members that exhibit variable levels of expression in different tissues [15]. The CNGC family is divided into five subfamily groups (I, II, III, IV-A and IV-B) based on their sequence similarity. Group I, II, and III are closely related, whereas groups IV-A and IV-B are distantly related to each other and to the other groups [29].

Heterologous expression systems and mutant plants have been used to characterize the functional roles of CNGC family members in ion transport across plant cells. Complementation analysis using K^+^- and Ca^2+^-uptake-deficient yeast mutants demonstrated that AtCNGC1 and AtCNGC2 partially recover K^+^ uptake [27], and that the AtCNGC11 and AtCNGC12 channels are permeable to Ca^2+^ [30]. Similarly, Leng et al. described AtCNGC2 as a CN-mediated cation channel that is permeable to Ca^2+^ and K^+^, but not Na^+^ [21,22]. When AtCNGC2 was introduced into *Xenopus laevis* oocytes lacking a low-affinity K^+^ uptake system or human embryonic kidney cells (HEK293), they displayed CN-dependent, inward-rectifying K^+^ currents and increased permeability to Ca^2+^ only in the presence of the CNs [21,22]. Electrophysiological studies of AtCNGC1 and AtCNGC4 suggested that they function in the uptake of monocations (such as K^+^ and Na^+^) [21,31]. In a reverse genetic study, a *atcngc2* knockout mutant exhibited hypersensitivity to Ca^2+^ but not to K^+^ or Na^+^ [32]. In addition, the *atcngc1* mutant also had a lower level of Ca^2+^ in shoots than wild-type plants [33]. *Atcngc3* mutant seedlings exhibited less sensitivity in their growth response to toxic levels of K^+^ and Na^+^ and lower cation levels in their tissues, relative to wild-type plants, indicating that AtCNGC3 plays a role in the non-selective uptake of monovalent cations [34]. *AtCNGC10* expression rescued the K^+^ uptake channel mutant, *akt1* (*Arabidopsis* Shaker-type K^+^ channel, AKT1), as well as similar *Escherichia coli* and yeast mutants, suggesting that *AtCNGC10* likely mediates K^+^ influx into cells. *AtCNGC10* overexpression results in 1.7-fold better growth of the *akt1* mutant under K^+^-limited conditions [35]. Although sequence similarity among the members of AtCNGC family ranges between 55% and 83% [29], they have exhibited discernible levels of specificity in the transport of diverse cations, and thus, the differences confer independent phenotypes with AtCNGC mutants. 

Due to their potential non-selectivity in the uptake of different cations, plant CNGCs have been predicted to also be involved in the uptake of micronutrient ions into cells, as well as toxic ions [34,36,37]. Nuclear magnetic resonance spectroscopy and chemical studies have been used to investigate the potential binding of Mg^2+^, Sr^2+^, or Pb^2+^ to Ca^2+^-binding sites of CaM in a distinct manner indicating an inhibitory effect on the interactions between CaM and functional targets, e.g., Ca^2+^ ion [38,39]. Interestingly, Pb^2+^ ion has been reported to enter into animal cells (bovine adrenal medullary cells or chromaffin cells) through Ca^2+^ channels [40,41]. Sunkar et al. reported that the expression of a truncated *NtCBP4* in transgenic tobacco and a mutation in *AtCNGC1* contribute to increased tolerance to Pb^2+^ [37]. In contrast, *NtCBP4* overexpression causes hypersensitivity to Pb^2+^ ion but tolerance to Ni^2+^ [26]. AtCNGC2 provides conductivity to other monovalent cations (Li^+^, Cs^+^, and Rb^+^), as high as to K^+^ ion, but much lower conductivity to Na^2+^ ion [21]. AtCNGC10 was also reported to regulate the transport of Mg^2+^ as well as Ca^2+^ ion in the shoots and roots of *Arabidopsis* plants. Based on studies utilizing heterologous expression and knockout systems, a consensus is developing that plant CNGCs can modulate the flux of a wide range of ions into plant cells. We have been interested in the relationship between members of the AtCNGC family and the uptake of diverse cations. In the current study, we examined the effect of two toxic heavy metal ions, Cd^2+^ and Pb^2+^, on different *Arabidopsis atcngc* mutant plants. Root growth and ion levels were analyzed to compare differences in the responses among the mutant lines and wild-type plants to the application of heavy metals. 

## 2. Results

### 2.1. Primary Root Growth Analysis in Pb^2+^ Stressed *atcngc* Mutant Plants

**Figure 1:**
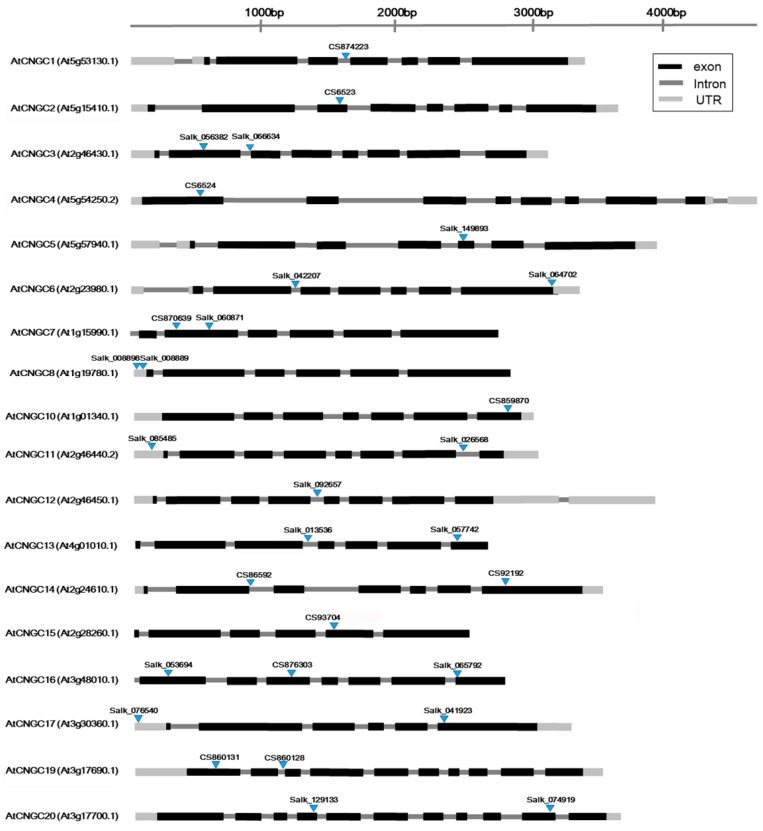
Schematic diagram illustrating the structure of the *Arabidopsis* CNGC family genes. The exon-intron structure in each of the genes is presented and the loci of the T-DNA insertions or single nucleotide substitutions (for *AtCNGC2*, *AtCNGC4*, *AtCNGC14* and *AtCNGC15*) in the *atcngc* knockout mutants are marked with upside-down triangles. The *Arabidopsis* mutant seeds were obtained from the Arabidopsis Biological Resource Center (ABRC) (https://abrc.osu.edu/): Light grey: UTR, dark grey: intron, black: exon.

**Figure 2:**
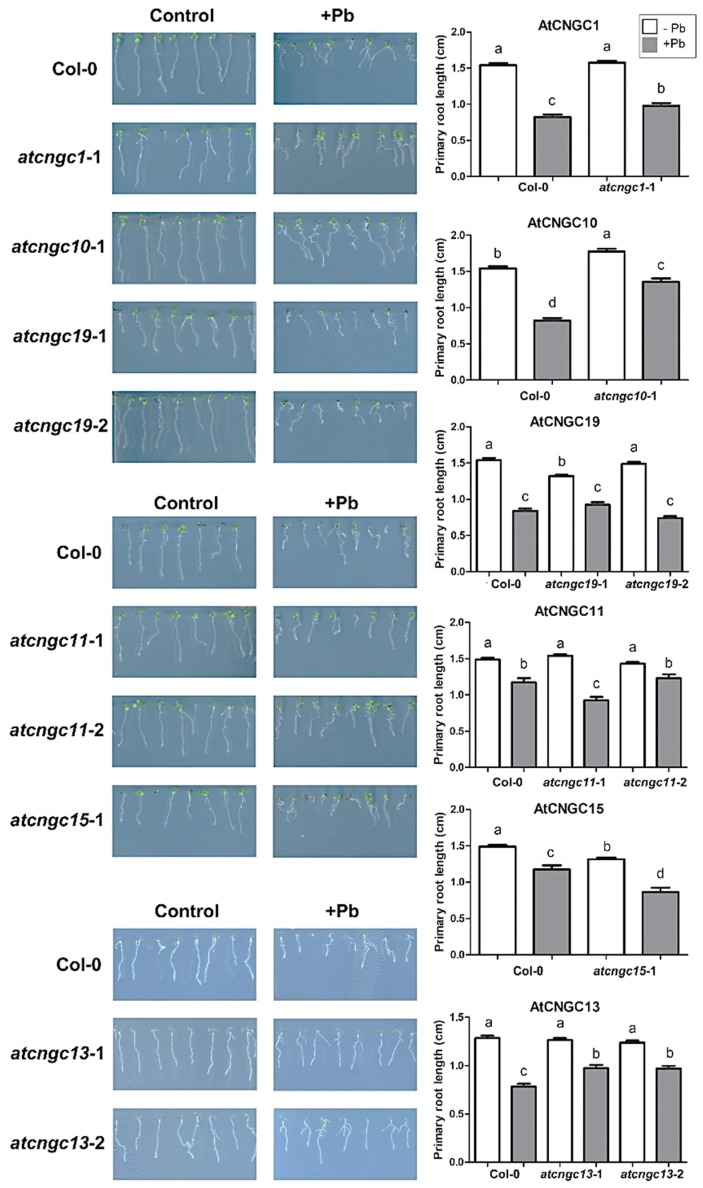
Growth of the *Arabidopsis* wild-type and *atcngc* mutants in response to Pb^2+^. Eight-day-old seedlings grown on agar media containing 150 μM Pb(NO_3_)_2_ were photographed and were used to measure primary root growth (length) using ImageJ software. The presented images (left side of figure) are representative of ≥80 seedlings of each genotype (the mutant or the wild-type) used to measure the primary root length for each treatment (right side of figure). Quantitative data presented represents the mean ± SEM. Data were subjected to a one-way ANOVA with a post-hoc Tukey’s comparison. Different letters indicate significant differences (*p* < 0.05).

The growth of primary roots in *Arabidopsis* mutants of all AtCNGC members, except AtCNGC9 and AtCNGC18, were measured to determine their response to heavy metal ions, relative to the response of Col-0 wild-type plants. One or more *Arabidopsis* mutation alleles for the eighteen distinct *AtCNGC* genes were used in this study (Figure 1). PCR assay and sequencing analysis were performed to clarify the genotypes and single nucleotide subsitution mutations, and some of PCR results present in Figure S1. The seeds of the mutant and wild-type plants used in this study were germinated and grown for eight days with or without 150 μM Pb(NO_3_)_2_. Then, the lengths of the primary root of plants were measured and compared between seedling plants grown on media with Pb^2+^ versus seedlings grown on media without Pb^2+^ ion. The results indicated that all of the eight-day-old seedlings grown on media with Pb^2+^ exhibited decreased primary root growth relative to roots of seedlings grown on media without Pb^2+^, although the original lengths of primary roots differed between different *Arabidopsis* mutants without the heavy metal (Figure 2). To more clearly define the level of root growth inhibition due to Pb^2+^, a finer measure of primary root length was made using ImageJ software, and statistical analyses of the effect of Pb^2+^ and differences between the mutant lines were conducted (Figure 2). Results indicated that the mutant lines of AtCNGC1 (CS874223, *atcngc1-*1), AtCNGC10 (CS859870, *atcngc10-*1), and AtCNGC13 (Salk_057742, *atcngc13-*1; Salk_013536, *atcngc13-*2) exhibited less growth inhibition in response to Pb^2+^, relative to the level of inhibition in the wild-type plants. In addition, one of the *AtCNGC19* mutant alleles, *atcngc19*-1, presented a shorter primary root compared to that of the wild-type or the other *AtCNGC19* mutant, *atcngc19-*2, in the absence of Pb^2+^, while the three different types of plant were grown with comparable growth size in the presence of Pb^2+^ (Figure 2). This relatively smaller growth reduction (root growth in +Pb^2+^/−Pb^2+^) of *atcngc19-*1 in the presence of Pb^2+^ indicates that the mutation in AtCNGC19 (CS860128, *atcngc19*-1) caused less negative effect on Pb^2+^ -stressed *Arabidopsis* plants. The other AtCNGC19 mutant allele (CS860131 *atcngc19*-2) showed a similar root length to the wild-type plants regardless of Pb^2+^ treatment. Mutants of the other AtCNGC members exhibited similar levels of growth inhibition to that of the wild-type (Figure S2). These data indicate that plants lacking *AtCNGC1*, *AtCNGC10*, *AtCNGC13,* or *AtCNGC19* exhibited increased tolerance to Pb^2+^ toxicity, and that these AtCNGC members are involved in Pb^2+^ uptake into plants. In contrast, plants lacking *AtCNGC15* (CS93704, *atcngc15-*1) or *AtCNGC11* (Salk_026568, *atcngc11-*1) exhibited increased inhibition of primary root growth relative to the wild-type. These data indicate that in contrast to the four previously described AtCNGC members, AtCNGC11 and AtCNGC15 function positively in regulating plant tolerance to Pb^2+^ stress (Figure 2). However, the other *AtCNGC11* mutant allele (Salk_085485, *atcngc11-*2) did not show significantly different root growth in response to Pb^2+^ compared to the wild-type. The Transfer-DNA (T-DNA) insertion in *atcngc11-*1 locates between the seventh exon and the eighth exon, whereas the insertion in *atcngc11-*2 locates in the 5’-UTR region. The different root growth responses to Pb^2+^ in *atcngc11* mutants may be due to the different locations of T-DNA insertions, but further confirmation is required.

### 2.2. Primary Root Growth Analysis in Cd^2+^ Stressed *atcngc* Mutant Plants

**Figure 3:**
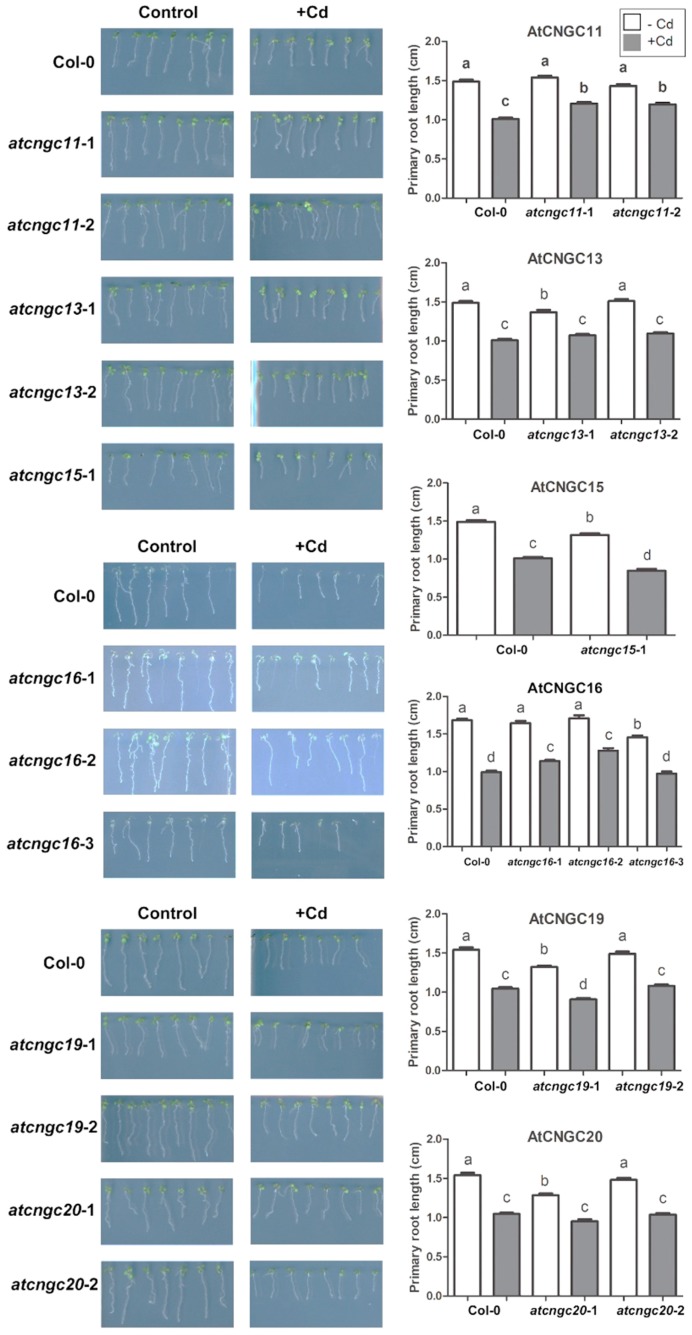
Growth of wild-type *Arabidopsis* and *atcngc* mutants in response to Cd^2+^. Eight-day-old seedlings grown on agar media supplemented with 55 μM CdCl_2_ were photographed and used to measure primary root growth (length) using ImageJ software. The presented images (left side of figure) are representatives of ≥80 seedlings of each genotype (the mutant or the wild-type) used to measure each treatment (right side of figure). Quantitative data presented represent the mean ± SEM. Data were subjected to a one-way ANOVA with a post-hoc Tukey’s comparison. Different letters indicate significant differences (*p* < 0.05).

Cadmium is also considered to be a very toxic heavy metal, with deleterious impacts on plant growth and the environment. Primary root length was also measured in eight-day-old wild-type and *atcngc* mutant lines grown on agar media with or without 55 μM CdCl_2_. The eight-day-old seedlings grown on agar media with Cd^2+^ appeared less healthy than seedlings grown without Cd^2+^ (Figure 3). Relative to the response of the wild-type plants, however, four different AtCNGC mutants were observed to have less growth retardation from the Cd^2+^ ion. Plants lacking AtCNGC11 (Salk_026568, *atcngc11-*1; Salk_085485, *atcngc11-*2), and AtCNGC16 (Salk_065792, *atcngc16-*1; Salk_053694, *atcngc16-*2) exhibited relatively less inhibition of primary root growth, while other AtCNGC mutants displayed similar levels of root growth inhibition as that of the wild-type (Figure 3 and Figure S3). Moreover, AtCNGC13 (Salk_057742, *atcngc13-*1) or AtCNGC20 (Salk_129133, *atcngc20-*1) have shorter primary roots relative to that of the wild-type in absence of Cd^2+^, however, the primary root lengths of *AtCNGC* mutants and the wild-type plants were comparable in presence of Cd^2+^, indicating that the less levels of growth inhibition in the *atcngc13-*1 and *atcngc20-*1 mutants by Cd^2+^ stress. The lower levels of primary root growth inhibition in the plants with the mutant alleles indicates the possibility that AtCNGC11, AtCNGC13, AtCNGC16, and AtCNGC20 function as positive components of Cd^2+^ uptake, similar to the result observed with Pb^2+^ (Figure 2). The mutant allele for AtCNGC13, *atcngc13-*1, displayed a lower level of primary root growth inhibition in response to both Cd^2+^ and Pb^2+^. These data suggest that AtCNGC13 functions negatively in the plant response to the two different heavy metal ion-contaminated conditions.

### 2.3. Quantitative Assessment of Pb^2+^ Levels in *atcngc* Mutant Plants

**Figure 4:**
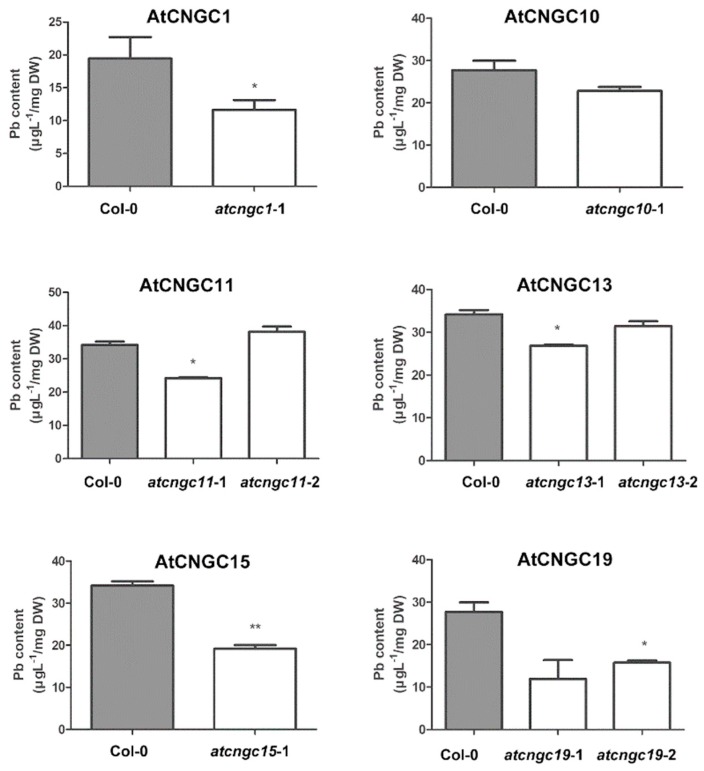
Pb^2+^ content in *Arabidopsis* plants grown on an agar medium with Pb^2+^. All of the seedlings used to measure primary root growth in three replicated experiments were combined and extracted in 60% (*v*/*v*) HNO_3_. Elemental analysis was conducted utilizing an ICP-MS System. Data presented represent the mean ± SEM (*n* = 3). T-tests were conducted to compare the concentration of each element in *cngc* mutants vs. wild-type plants. * and ** symbols indicate significant differences at *p* > 0.05 and *p* > 0.01, respectively.

All of the *Arabidopsis* plantlets that were used to measure primary root length were collected and analyzed for heavy metal ion content to determine if the alteration in growth response to Pb^2+^ in the *atcngc* mutants was related to differential accumulation of heavy metals between the wild-type and mutant plants. The contents of the Ca^2+^, K^+^, Pb^2+^ and Cd^2+^ ions were determined using an ICP-MS system, and the final value was calculated based on dry weight. K^+^ levels in the wild-type system, and the final value was calculated based on the dry weight. K^+^ levels in the wild-type versus *atcngc* mutant plantlets were not significantly different among the wild-type and AtCNGC mutants, although the Ca^2+^ content in some *atcngc* mutant lines was slightly lower than that in the wild-type (Figure S4). Consistent with the phenotypic analysis presented in Figure 2, a lower concentration of Pb^2+^ ion was found in the *atcngc1*-1, *atcngc13*-1, and *atcngc19*-1 mutants, but not in the *atcngc10*-1 mutant, than in the wild-type plants (Figure 4); indicating that the lower level of primary root growth inhibition was, at least in part, the result of a lower accumulation of Pb^2+^ in the mutant plants. The *atcngc13*-1 and *atcngc19*-1 mutant plants, however, exhibited less and similar growth retardation, respectively, to the wild-type; while Pb^2+^ ion accumulation in the *atcngc13*-2 mutant and *atcngc19*-2 mutant, respectively, was either comparable or lower than what was found in the wild-type plants (Figure 2 and Figure 4). Additionally, *atcngc11*-1 and *atcngc15*-1 mutants also had lower levels of Pb^2+^ than the wild-type plants, despite exhibiting a higher level of primary root growth inhibition than the wild-type plants when grown in the presence of Pb^2+^ (Figure 2 and Figure 4). This indicated that AtCNGC11 or AtCNGC15 have possible roles in Pb^2+^ ion uptake, but the further inhibition of root growth in their mutants by Pb^2+^ treatment may not directly result from a lower Pb^2+^ content in the mutant plants. Collectively, the quantitative analysis of heavy metal content confirmed the relationship between the growth defect phenotype and reduced Pb^2+^ ion levels in *Arabidopsis atcngc1*, *atcngc13,* and *atcngc19* seedlings. In addition, these data indicate that at least AtCNGC13 and AtCNGC19 as well as AtCNGC1 appear to be involved in Pb^2+^ ion uptake into plant cells. These results are consistent with a previous study on AtCNGC1-related Pb^2+^ ion uptake [37].

### 2.4. Quantitative Assessment of Cd^2+^ Levels in *atcngc* Mutant Plants

**Figure 5:**
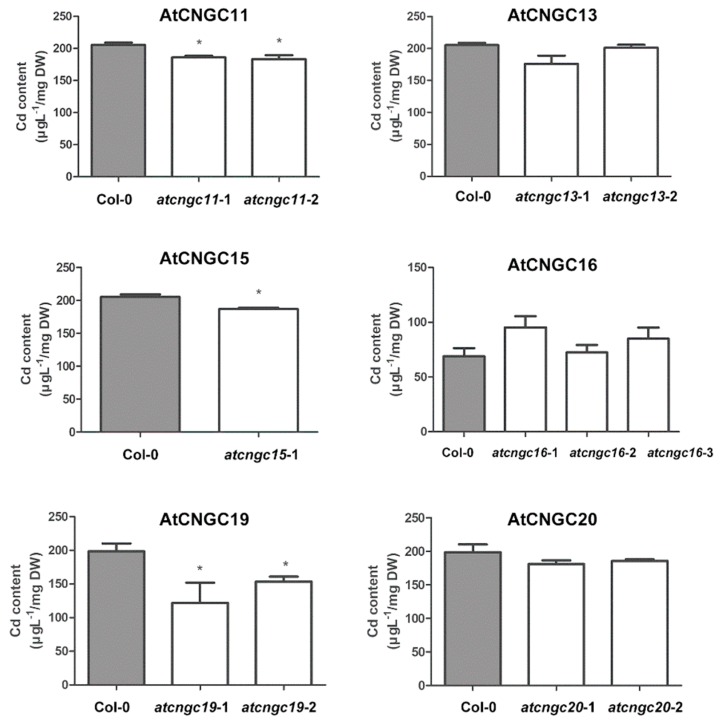
Cd^2+^ content in *Arabidopsis* plants grown on an agar medium with Cd^2+^. All of the seedlings used to measure primary root growth in three replicated experiments were combined and extracted in 60% (*v*/*v*) HNO_3_. Elemental analysis was conducted utilizing an ICP-MS System. Data presented represent the mean ± SEM (*n* = 3). T-tests were conducted to compare the concentration of each element in *cngc* mutants vs. wild-type plants. * and ** symbols indicate significant differences at *p* > 0.05 and *p* > 0.01, respectively.

Cd^2+^ content was also assessed in the eight-day-old mutant and wild-type plantlets grown in the presence of Cd^2+^ ion that were used to measure primary root length. Among the *atcngc* mutants that exhibited a lower inhibition of primary root growth, Cd^2+^ ion accumulation was lower only in the *atcngc11-*1, and *atcngc11-*2 mutants than it was in the wild-type plantlets; while the Cd^2+^ levels in the *atcngc13-*1 and *atcngc20-*1 mutants and the three *atcngc16* mutants were not significantly different than the level found in the wild-type plants (Figure 3 and Figure 5). Taken together, it is probable that AtCNGC11 has a role in Cd^2+^ ion entry into plants, and thus negatively affects plant tolerance to Cd^2+^ stress. Notably, *atcngc15-*1, as well as *atcngc19-*1 and *atcngc19-*2, mutants exhibited less Cd^2+^ ion accumulation in the plantlets, although no differences in the level of primary root growth inhibition relative to wild-type plants were observed. This may be due to their shorter primary root lengths than those of wild-type plants even without Cd^2+^ treatment. These data suggest that AtCNGC15 and AtCNGC19 may have some relationship to Cd^2+^ ion uptake, but also in the regulation of root development resulting from Cd^2+^ contamination. Collectively, the data indicate that AtCNGC11 and possibly AtCNGC15 and AtCNGC19 are potential components of Cd^2+^ ion uptake into plant cells. 

## 3. Discussion

The CNGC proteins are typically known to Ca^2+^-permeable channels involved in the uptake of diverse monovalent cations and several CNGC members also display the ability to mediate the transport of divalent cations [21,22,30,31,34,36]. Apart from a study on the positive participation of AtCNGC1 in Pb^2+^ ion uptake into plants, relatively little information exists on the role of *Arabidopsis* AtCNGC members in the uptake of toxic heavy metal ions. In the present study, the potential role of the various *Arabidopsis* CNGC family members in ion uptake was investigated, especially the heavy metal Pb^2+^ and Cd^2+^ ions by utilizing a series of *atcngc* mutants. The results indicated that the knockout of specific *AtCNGC* genes confers tolerance to Pb^2+^ or Cd^2+^ ion as measured by lower inhibition of primary root growth. Lower inhibition of primary root growth phenotypes with less accumulation of heavy metal ions was observed in several *atcngc* mutants. The results indicated that AtCNGC1, AtCNGC10, AtCNGC13, and AtCNGC19 have negative effects on Pb^2+^ stress, while AtCNGC11, AtCNGC13, AtCNGC16, and AtCNGC20 have negative effects on Cd^2+^ stress. Therefore, AtCNGC13 functions as a negative component in plant tolerance to both Pb^2+^ and Cd^2^_._ Conversely, AtCNGC11 and AtCNGC15 appear to be positively involved in plant tolerance to Pb^2+^ in *Arabidopsis* (Figure 2 and Figure 3).

The analysis of heavy metal content indicated that AtCNGC11 and AtCNGC15 as well as AtCNGC1, AtCNGC13, and AtCNGC19 are potential factors in the uptake mechanism of Pb^2+^ ion into plant cells, while AtCNGC11, AtCNGC15, and AtCNGC19 are likely involved in Cd^2+^ uptake (Figure 4 and Figure 5). Unlike AtCNGC1, AtCNGC13, or AtCNGC19, the mutation of either AtCNGC11 (*atcngc11-*1) or AtCNGC15 (*atcngc15-*1) confers less tolerance to Pb^2+^ stress than the wild-type (Figure 2). Differences in the obtained results with the various AtCNGC members may be partially due to the ability of AtCNGC11 or AtCNGC15 to indirectly activate the other AtCNGCs or other ion transport channels that compensate for the lack of the ions in plants. Thus, the activated channels cause a more severe phenotype by enhancing toxic ion uptake. This speculation, however, needs to be further investigated with additional molecular and biological studies. To the best of our knowledge, the present study is the first to identify several AtCNGC members as potential components in the plant response to the heavy metal ions Pb^2+^ and Cd^2+^ and, more specifically, the uptake of Pb^2+^ and Cd^2+^ ions. Importantly, while knockout of *AtCNGC10* rendered *Arabidopsis* plants more tolerant to Pb^2+^, and knockout of *AtCNGC13*, *AtCNGC16,* or *AtCNGC20* resulted in greater tolerance to Cd^2+^ treatment, respectively, no significant difference in heavy metal contents was confirmed in the mutant plants relative to the wild-type plants. This discrepancy between the plant root growth and the heavy ions content in the four different *AtCNGC* mutants indicates that these specific AtCNGC members are not directly involved in Cd^2+^ or Pb^2+^ ion uptake into plant cells but rather, function in a negative manner in regard to plant tolerance to heavy metals.

In the present study, there was a discrepancy in the phenotypic and heavy metal ion content results obtained using two different mutation alleles of the same *AtCNGC* genes (e.g., *atcngc13-*1, *atcngc13-*2 on Pb^2+^ and Cd^2+^ stress). AtCNGC members are known to be activated through the binding of signal molecules, such as cyclic nucleotides, on CNBDs; however, the different responses to Pb^2+^ and/or Cd^2+^ in mutants of the same AtCNGCs (e.g., *atcngc13-*1 and *atcngc13-*2) are likely due to the different locations of the T-DNA insertion (Figure 1). However, the obvious developmental defects of the *AtCNGC* mutants have not been revealed except for the *atcngc2* and *atcngc4* mutants showing severe growth phenotype and infertility [42]. Despite some mutant lines exhibiting a similar level of inhibition of primary root growth in response to Pb^2+^ and Cd^2+^ as the wild-type plants, most of the *AtCNGC* mutants accumulated fewer heavy metals. This strongly suggests that AtCNGC members play a functional role in the uptake process for Cd^2+^ or Pb^2+^ ions.

**Figure 6:**
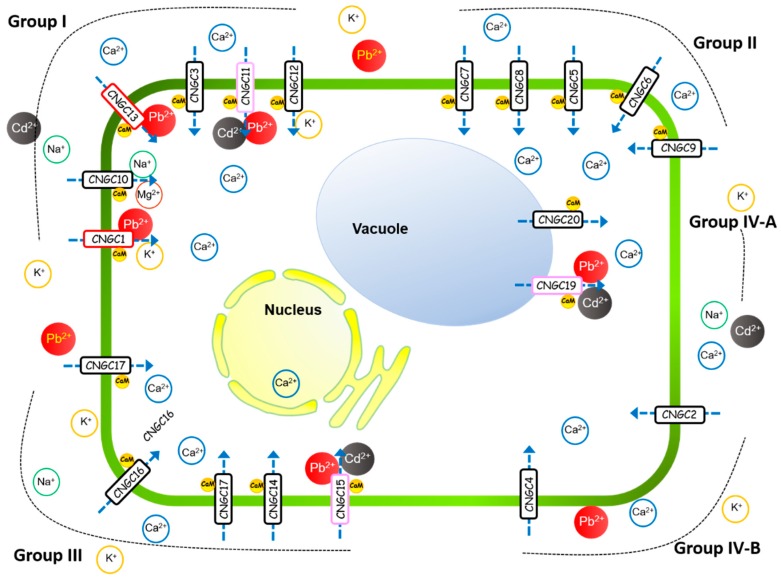
Model of the involvement of AtCNGC family members in ion transport in plants. AtCNGC members are divided in five groups according to the alignment of their predicted amino acid sequences, and locate to the plasma membrane (group I, II, III, IV-B) or vacuolar membranes (IV-A) (drawn as adjacent shapes with arrows) [12,29]. Ions are transported with the help of Ca^2+^-permeable AtCNGC family members dependent on the different ions. AtCNGCs in red rectangle: for Pb^2+^ entry, AtCNGCs in pink rectangle: for Pb^2+^ or Cd^2+^ entry.

Twenty *Arabidopsis* CNGC members share 55% to 83% sequence similarity, and are divided into five evolutionary groups based on the alignment of the predicted amino acid sequences. These subclasses are designated as groups I, II, III, IV-A, and IV-B [29]. In the present study, among the AtCNGC members proposed to function in Pb^2+^ ion uptake, AtCNGC1, AtCNGC11, and AtCNGC13 are in group I, while AtCNGC15 and AtCNGC19 belong to group III and IV-A, respectively (Figure 6). Interestingly, NtCBP4, a tobacco CNGC, has also been reported to be a component of Pb^2+^ ion uptake in plants, and is closely related to AtCNGC1 based on the alignment of their protein sequences [29,37]. Since AtCNGC and the tobacco NtCBP4 have been suggested to share a common ancestor [29], AtCNGC11 and AtCNGC13 may have also been derived from the same ancestor as NtCBP4. As group I also includes AtCNGC11, which has also been implicated as a potential transporter of Cd^2+^, other AtCNGC members may have potential roles as components of Cd^2+^ transport. AtCNGC16 and AtCNGC20, which exhibit a negative relationship to the plant tolerance response to Cd^2+^, are members of group II and IV-A, respectively (Figure 6). Notably, none of the AtCNGCs in group II and IV-B have been identified as components of plant tolerance to heavy metals or heavy metal ion uptake. This suggests that the AtCNGCs in these distinct groups have different characteristics in regard to their roles in heavy metal uptake. 

*Arabidopsis* CNGCs have been generally hypothesized to localize to the plasma membrane, although AtCNGC19 and AtCNGC20 have been previously reported to localize to the tonoplast; suggesting that they function as passive transporters of cations between the vacuole and cytosol [12,43]. Toxic ions are preferentially sequestered into the vacuole to counteract their toxicity in the cytosol [44]. In previous studies, genes encoding AtCNGC19 and AtCNGC20 were upregulated in response to salinity stress and it was suggested that AtCNGC19 and AtCNGC20 function to mediate the plant response to salt stress by mediating Ca^2+^ signaling [43,45,46]. Despite the data in the present study indicating a negative role for AtCNGC20 in plant tolerance response to Cd^2+^, it was suggested that CNGC19 and CNGC20 play roles in the plant response to toxic heavy metal ions, including Cd^2+^.

In the present study, we found that several members of the *Arabidopsis* CNGC family have potential roles in plant tolerance to heavy metals and uptake of Pb^2+^ and Cd^2+^. As non-selective cation transporters, CNGCs represent possible entry pathways for heavy metal ions; however, only a few cyclic nucleotide target proteins have been connected to this process [26,37]. The results of the current study indicate that a number of plant CNGC members are potentially involved in heavy metal uptake and tolerance in plants. 

## 4. Materials and Methods

### 4.1. Plant Material and Growth Condition

*Arabidopsis thaliana* L. (Heynh) ecotype Columbia-0 (Col-0) was used in the study, along with a series of T–DNA insertion or single nucleotide substitution mutants for AtCNGCs. Seeds for the mutant lines were obtained from the Arabidopsis Biological Resource Center (ABRC) (https://abrc.osu.edu/). The selected mutant lines were: AtCNGC1 (CS874223, *atcngc1-*1), AtCNGC2 (CS6523), AtCNGC3 (Salk_056832; Salk_066634), AtCNGC4 (CS6524), AtCNGC5 (Salk_149893), AtCNGC6 (Salk_064702; Salk_042207), AtCNGC7 (Salk_060871; CS870639), AtCNGC8 (Salk_008889; Salk_008898), AtCNGC10 (CS859870, *atcngc10-*1), AtCNGC11 (Salk_026568, *atcngc11-*1; Salk_085485, *atcngc11-*2) AtCNGC12 (Salk_092657), AtCNGC13 (Salk_057742, *atcngc13-*1; Salk_013536, *atcngc13-*2), AtCNGC14 (CS86592; CS92192), AtCNGC15 (CS93704, *atcngc15-*1), AtCNGC16 (Salk_065792, *atcngc16-*1; Salk_053694, *atcngc16-*2; CS876303, *atcngc16-*3), AtCNGC17 (Salk_041923; Salk_076540), AtCNGC19 (CS860128, *atcngc19-*1; CS860131, *atcngc19-*2), AtCNGC20 (Salk_129133, *atcngc20-*1; Salk_074919, *atcngc20-*2) in the Col-0 background. The seeds of all plant lines were surface sterilized with 70% (*v*/*v*) ethanol and 0.05% (*v*/*v*) Triton X-100, then rinsed in Milli-Q water and placed on media containing 1.75 mM KCl, 50 μM H_3_BO_3_, 10 μM MnCl_2_, 2 μM ZnSO_4_, 1.5 μM CuSO_4_, 0.075 μM NH_4_Mo_7_O_24_, 74 μM Fe-EDTA, 0.5 mM phosphoric acid, 2 mM Ca(NO_3_)_2_, and 0.75 mM MgSO_4_ at pH 5.8 with Ca(OH)_2_, 1% (*w*/*v*) sucrose, and 1% (*w*/*v*) Agarose L03 (TAKARA, Kusatsu, Japan). Media with 150 μM Pb(NO_3_)_2_, or 55 μM CdCl_2_·2.5H_2_O were used for applying heavy metal stress. After stratification for 3 days at 4 ℃, seeds were germinated and grown on vertically positioned plates in a controlled growth cabinet for 8 days, with a 16/8 h light/dark cycle (80 to 100 μmol m^−2^ s^−1^) at 22 °C. 

### 4.2. Root Growth Assay

Plants grown for 8 days were photographed and the lengths of the primary roots (≥80 seedling plants of each genotype studied for each treatment) were analyzed using ImageJ software (National Institutes of Health, Bethesda, MD, USA). All experiments were performed three times, and a representative set of data are presented. Statistical analysis consisted of a one-way ANOVA with a post-hoc Tukey’s comparison using GraphPad Prism (GraphPad Software, San Diego, CA, USA) software. 

### 4.3. Ion Content

Three biological replicates consisting of 30–40 pooled seedlings in each replicate were analyzed. Whole eight-day-old plantlets were harvested, rinsed in Milli-Q water, and dried in an oven at 65 °C for 4 days. Two ± 0.1 mg of dried samples were degraded in 1 mL of 60% (*v*/*v*) HNO_3_ by heating at 125 °C for 3 h. The resulting samples were then diluted with Milli-Q water to 10 mL. Elemental levels were measured by inductively coupled plasma mass spectrometry (NexION^®^ 300 ICP-MS System, Perkin Elmer, Waltham, MA, USA), and concentrations of the elements were calculated based on dry weight. Statistical differences were evaluated with a *T*-test using GraphPad Prism software (GraphPad Software, San Diego, CA, USA). 

## Supplementary Materials

Supplementary materials can be found at http://www.mdpi.com/1422-0067/20/2/413/s1.

## Abbreviations

CNGC	Cyclic Nucleotide-Gated Channel
CN	Cyclic Nucleotide
cAMP	cyclic Adenosine MonoPhosphate
cGMP	cyclic Guanosine MonoPhosphate
CNBD	CN binding domains
CaM	Ca^2+^-activated calmodulin
ROS	Reactive Oxygen Species
