# Supplementary material for: Arabidopsis CNGC Family Members Contribute to Heavy Metal Ion Uptake in Plants
Source: Int J Mol Sci. 2019 Jan 18;20(2):413. doi: 10.3390/ijms20020413 (PMC6358908; doi:10.3390/ijms20020413)
Supplement: Supplementary file 1 [file ijms-20-00413-s001.pdf]

International Journal of
Molecular Sciences

MDPI

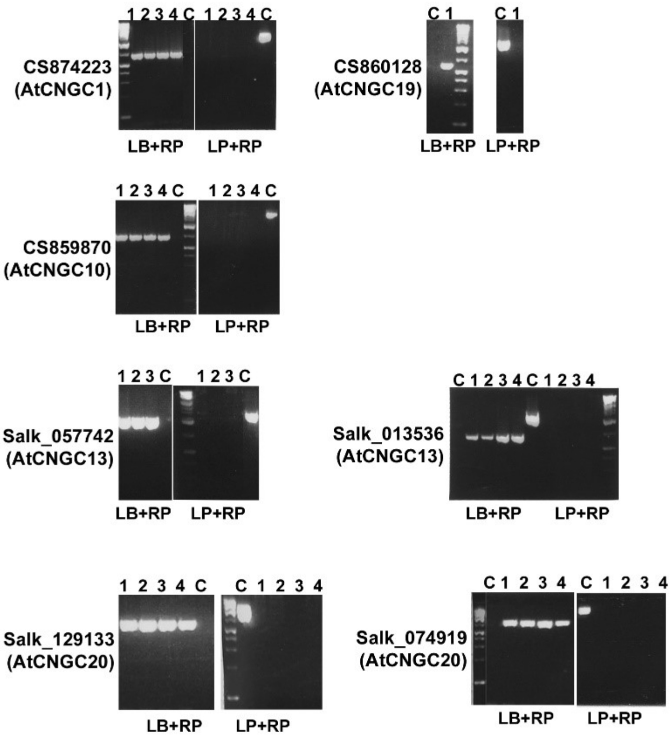

Figure S1. Genotyping of the T-DNA inserted *AtCNGC* mutants. Genomic DNAs were prepared with rosette leaf-tissues of *Arabidopsis* wild-type (Col-0, C) and *atcngc* mutants (numbered). Genotyping PCR assays were performed using the genomic DNAs with sequence-specific primers (LP and RP) and LB1.3 (LB) primers that were provided in Salk Institute Genomic Analysis Laboratory (http://signal.salk.edu/ tdnaprimers.2.html).

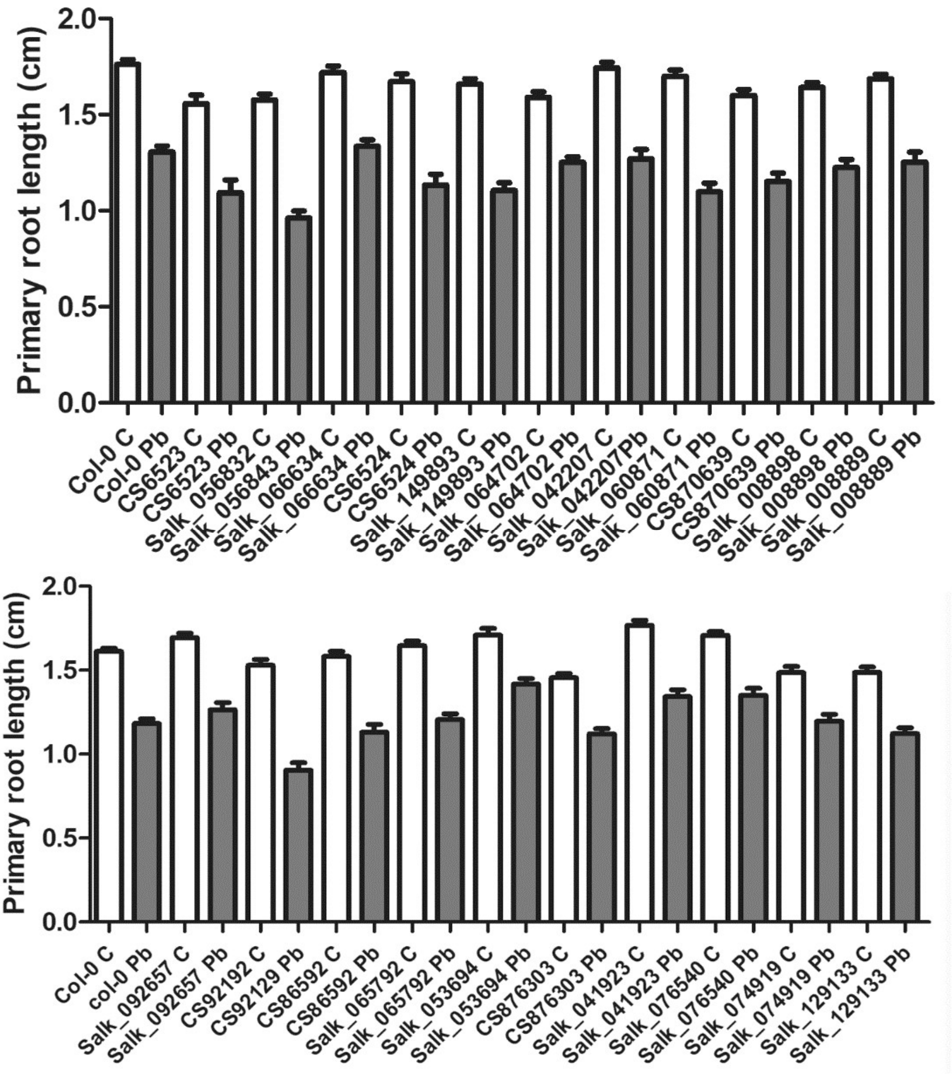

Figure S2. Growth of *Arabidopsis atcngc* mutants on an agar medium containing Pb^{2+}. *Arabidopsis* wild-type and *atcngc* mutants were grown for eight days on vertical agar medium plates containing 150 μM $Pb(NO_3)_2$. The values presented represent the mean ± se of primary root length (n = ≥ 80 plantlets per treatment from three replicated experiments). C: no Pb^{2+} treatment

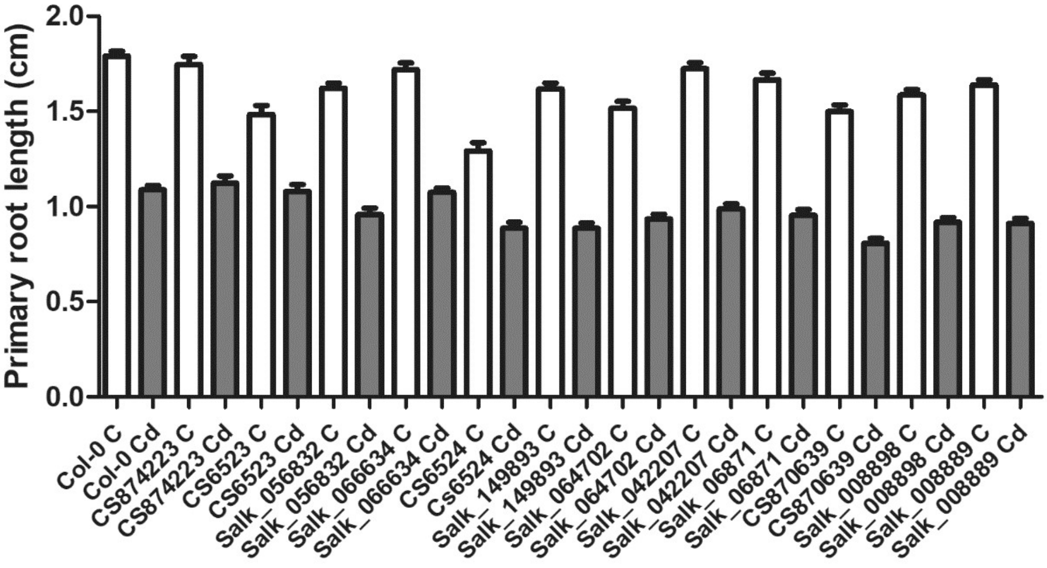

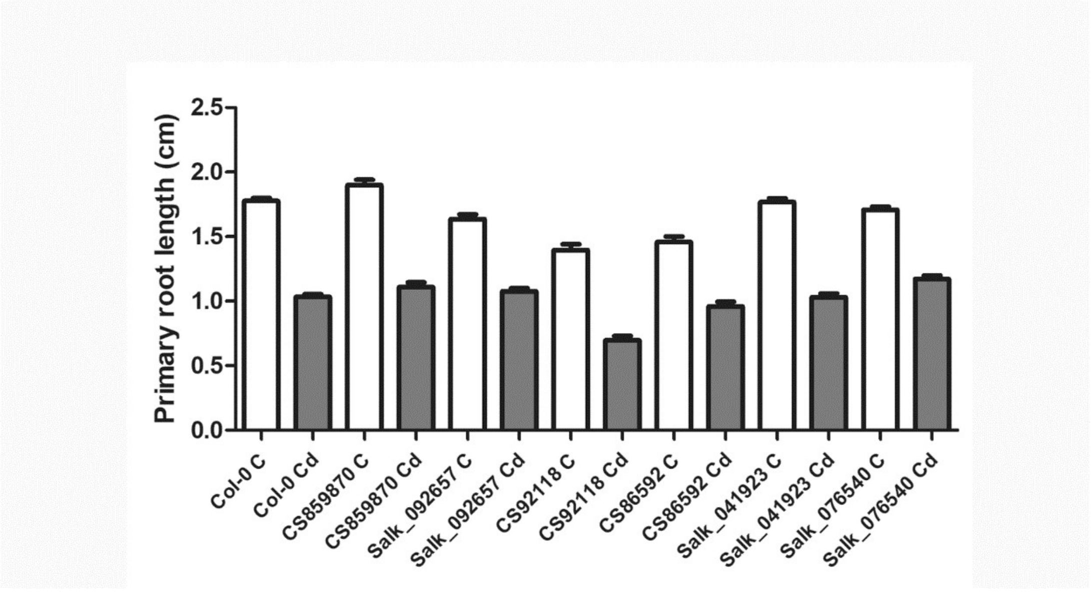

Figure S3. Growth of *Arabidopsis atcngc* mutants on an agar medium containing Cd^{2+}. *Arabidopsis* wild-type (Col-0) and *atcngc* mutants were grown for eight days on vertical agar medium plates containing 55 μM $CdCl_2 \cdot 2.5H_2O$. The values presented represent the mean ± se of primary root length (n = ≥ 80 plantlets per treatment from three replicated experiments). C: no Cd^{2+} treatment

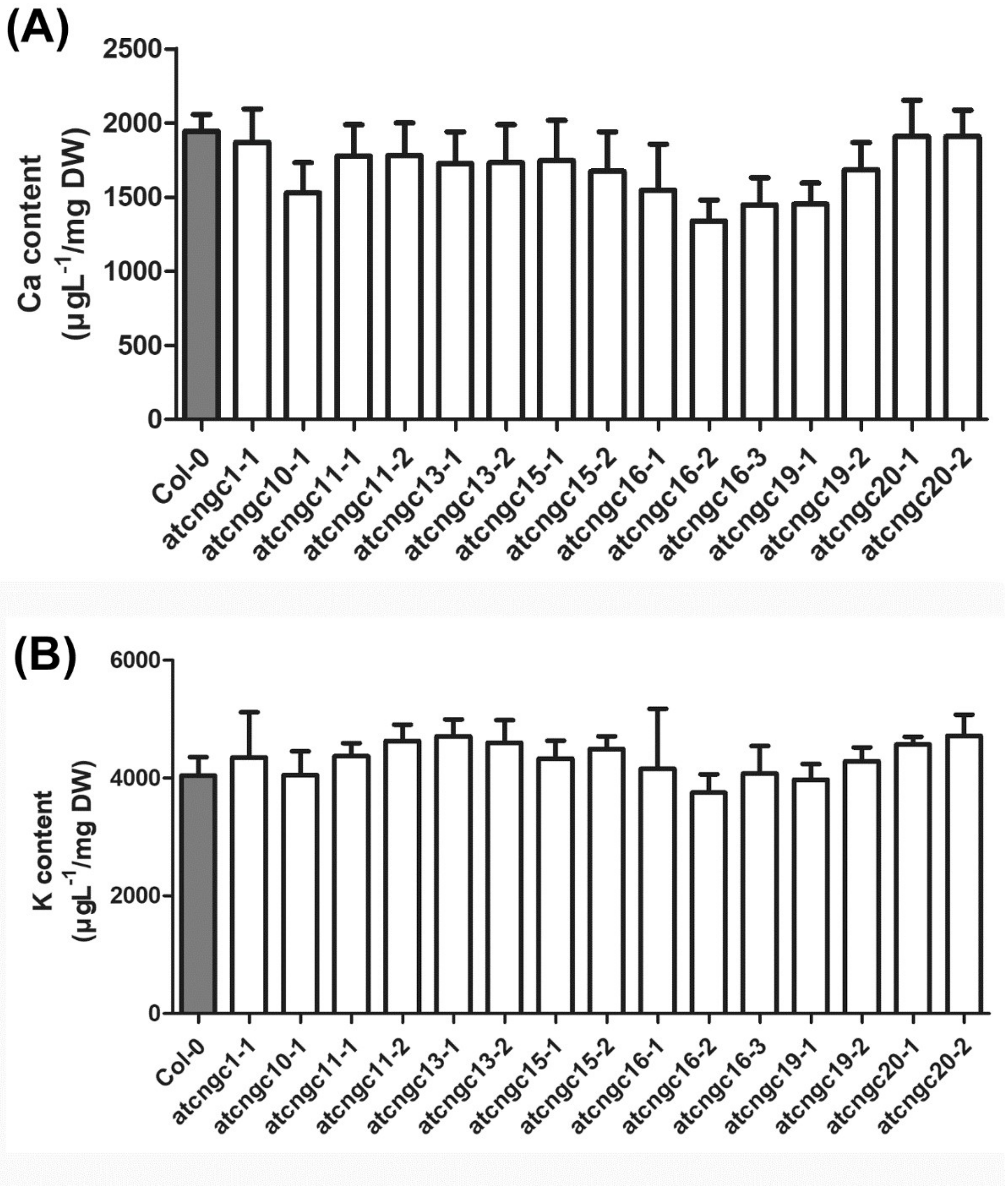

Figure S4. Ca^{2+} and K^{+} content in the *Arabidopsis* wild-type and *atcngc* mutants. Eight-day-old plantlets grown on media without heavy metal ions were collected from individuals from three replicated experiments and extracted in 60% (v/v) HNO_3. Elemental analysis was conducted with an ICP-MS System. Data presented represent the mean ± se (n = 3).
